# Relationships between personal human values and social value orientation

**DOI:** 10.1371/journal.pone.0312795

**Published:** 2024-11-27

**Authors:** John B. Nezlek

**Affiliations:** 1 Center for Climate Action and Social Transformations (4CAST), Institute of Psychology, SWPS University, Warsaw, Poland; 2 Department of Psychological Sciences, College of William & Mary, Williamsburg, Virginia, United States of America; Georg-August-Universität Göttingen: Georg-August-Universitat Gottingen, GERMANY

## Abstract

The present study examined relationships between social value orientation and personal values. Participants, n = 1655, were university students (*M*_age_ = 18.9 years, 60% women) who completed the Triple Dominance Measure, a measure of social value orientation, and the PVQ-21, a measure of Schwartz’s personal values. Two items were added to the PVQ-21 to measure benevolence toward people in general. The analyses found that pro-socials had significantly higher scores than pro-selfs (Competitors and Individualists) for Benevolence, Universalism, and Conformity values, whereas pro-selfs had significantly higher scores than pro-socials on Hedonic, Achievement, and Power values. These differences reflected the fact that Benevolence, Universalism, and Conformity values concern the feelings and well-being of others, concerns that are consistent with a pro-social orientation. In contrast, Hedonic, Achievement, and Power values concern self-enhancement, concerns that are consistent with a self-focused orientation. There were no significant differences between individualists and competitors for any value, nor were there differences of any kind for Tradition, Security, Self-direction, and Stimulation values. These results complement and expand previous research by demonstrating how individual differences in social value orientation are related to individual differences in fundamental, personal human values.

## Introduction

The present study was designed to add to existing research on relationships between social value orientation (SVO) and values conceptualized more broadly. Although the word value is part of SVO, there is little research on relationships between SVO and values understood more broadly. The term “social value” refers to how much people value others versus how much they value themselves, and it does not refer to personal values conceptualized in terms of people’s beliefs about right and wrong, good and bad, and so forth. Nevertheless, there are good reasons to believe that personal values and SVO should be related, and I discuss these reasons below.

The present study was motivated by a desire to provide a better understanding of social value orientation. As explained below, SVO is typically inferred from a series of behavioral choices. People receive scores or are classified into categories that represent the extent to which and how they balance an emphasis on their own well-being with an emphasis on the well-being of others.

As behavioral measures, measures of SVO do not provide direct information about the psychological processes that underlie or are responsible for the behaviors that are used to measure SVO. To understand such processes, researchers have examined relationships between SVO and personality [1, 2], between SVO and politics [3], between SVO and various prosocial behaviors such as donations [4], and other constructs. Although each of these bodies of research has provided insights into the nature of SVO, I believe that our present understanding of SVO can be increased by understanding the relationships between SVO and personal values.

### Social value orientation

Social value orientation refers to how people balance the competing interests of rewarding the self and rewarding others. An important impetus for research on SVO was the recognition that people are motivated by more than narrow self-interest, what Ryan and Ackermann [5] described as “Homo economicus.” People do not think only of themselves, although there is considerable variability in the extent to which they take the benefits others receive into account and exactly how this is done. These differences are the focus of measures of SVO.

Unlike many individual difference measures that rely on self-reports of attitudes, beliefs, and self-evaluations, SVO is typically measured with some type of behavior. For example, over a series of trials, individuals allocate rewards to themselves and to partners in what are usually called economic games. The rewards are typically money or points, and the games vary in terms of the specific type of choice, sometimes referred to as a social dilemma, that is measured. The games consist of a series of choices, and a person’s social value orientation is inferred from the choices he or she makes in these games.

Although SVO can be described in various ways, the distinction that is emphasized in much of the research on SVO is the distinction between pro-social and pro-self. Pro-social refers to an orientation that reflects taking rewards to the self and to others into account when making allocation decisions, whereas pro-self refers to an orientation that reflects taking only rewards to the self into account when making allocation decisions. SVO is frequently operationalized in terms of categories, and individuals can be classified as pro-social or pro-self.

Although there have been concerns about measuring SVO as a category [5], category-based measures of SVO such as the Triple Dominance Measure [TDM; 6] have been found to be related to various individual differences outside of cooperation and resource allocation. For example, Van Lange et al., [7] found that pro-selfs were more likely to vote for conservative candidates than prosocials. Similarly, Chirumbolo et al. [3] found that pro-selfs were more right-wing and were more authoritarian than prosocials. In their review of individual differences in prosocial behavior as measured by economic games, Thielmann et al. [2] found that prosociality was positively related to Agreeableness, Openness, and the Honesty/Humility factor of the HEXACO model. In other words, although SVO is measured within the context of economic games, the individual differences it measures are relevant to understanding a potentially broad range of phenomena.

In the present study, SVO was measured using the TDM. The TDM provides a basis for classifying people into one of three categories: pro-socials, individualists, and competitors. The last two categories are frequently combined into a single category labeled pro-selfs, and in the present study, differences among the three categories, differences between pro-selfs and pro-socials, and differences between the two types of pro-self orientation (individualists and competitors) were examined.

### Personal values

In contrast to social value orientation, which focuses on the specifics of social exchange, personal values concern a much broader range of phenomena. As proposed by Schwartz [8]: (1) Values are beliefs about the importance of desirable goals, (2) When activated, values elicit emotion, (3) Values are basic goals that apply across specific situations, (4) Values consciously or unconsciously motivate behavior, perception, and attitudes, (5) Value effects occur through a process of trade-offs among the relevant values, (6) Values serve as standards for evaluating actions, people, and events, and (7) Values are ordered by importance in a relatively enduring hierarchical system. Schwartz’s model of personal values, like that of Rokeach [9] and other theorists, concerns how people think of their relationships with world at large, their sense of what is important to them, and ultimately, what motivates their behavior and provides a rationale for their beliefs.

Schwartz posited that there are ten basic personal values: Universalism, Benevolence, Conformity, Tradition, Security, Self-direction, Stimulation, Hedonism, Achievement, and Power. The validity of this model has been demonstrated numerous times, including large scale cross-cultural studies [e.g., 10]. Brief descriptions of these 10 values, taken from Schwartz and Cieciuch [10], are presented in Table 1.

**Table 1:** Definitions of Schwartz’s basic personal values with additional measure of Benevolence in general.

Benevolence	Preservation and enhancement of the welfare of people with whom one is in frequent personal contact
Universalism	Understanding, appreciation, tolerance, and protection for the welfare of all people and of nature
Conformity	The restraint of actions, inclinations, and impulses that are likely to upset or harm others and violate social expectations or norms
Tradition	Respect, commitment, and acceptance of the customs and ideas that traditional culture or religion provides
Security	Safety, harmony, and stability of society, relationships, and self
Stimulation	Excitement, novelty, and challenge in life
Self-Direction	Independent thought and action, choosing, creating, and exploring
Achievement	Personal success through demonstrating competence according to social standards
Hedonism	Pleasure and sensuous gratification for oneself
Power	Control or dominance over people and resources
Benevolence-General	Preservation and enhancement of the welfare of people in general.

The descriptions in Table 1 also include a new measure of Benevolence that was created for this study. This new measure concerns benevolence towards people in general, as opposed to benevolence to close others, which is the focus of Schwartz’s measure. Within Schwartz’s model, benevolence is defined as “Preservation and enhancement of the welfare of people with whom one is in frequent personal contact.” The focus of Benevolence on close others is evident in the two items from Schwartz’s scale that measure Benevolence: “It is very important to him to help the people around him. He wants to care for their well being,” and “It is important to her to be loyal to her friends; She wants to devote herself to people close to her.”

The new items of benevolence refer to the same constructs, care and trustworthiness, but they concern the care of and goodwill toward those beyond someone’s immediate contacts or close others. I believed this new measure was needed because I believed it was necessary to have a measure of benevolence that was not limited to people whom the respondent knew. None of the measures of other values in Schwartz’s model specify close others (in any way). If other people are mentioned, it is in general terms, e.g., an item measuring Power reads: “It is important to her to get respect for others. She wants people to do what she says.”

Measuring benevolence in terms of the “generalized other” makes the measurement level of benevolence the same as measurement level of the other values in Schwartz’s system. This new construct was measured with two items that corresponded to the focus of the two original items. One item concerned care: “It is important to him to be kind and caring to people, whether he knows them or not,” and the other item concerned trust: “It is important to her that other people trust and can rely upon her. She people to know that she is trustworthy.” As explained in the Methods section, items in Schwartz’s measure are gender specific.

Measuring benevolence in terms of the generalized other also provided a level of measurement that corresponded to the level of measurement of SVO. Unless specified otherwise, as in the case of an experiment with a specific partner of some kind, measures of SVO concern the allocation of rewards to an anonymous person. In terms of the present study, the TDM measure of SVO asks about reward allocation to a hypothetical person. This means that the focus (or level of analysis) of Schwartz’s original measure of Benevolence and the focus of the TDM were meaningfully different: close others for Benevolence vs. anonymous (presumably everyone) for the TDM. Differences between the levels of analysis of the measures of SVO and Benevolence could compromise the validity of analyses examining relationships between SVO and Benevolence.

Regardless of the level of measurement of Benevolence, it is critical to distinguish the Schwartz factors of Universalism and Benevolence. Although scores measuring these two basic values are often combined to form a score on higher-order factor, typically labelled as Self-transcendence, such a higher-order scale score can obscure differences between Universalism and Benevolence. Universalism is defined as “Understanding, appreciation, tolerance, and protection for the welfare of all people and of nature,” e.g., Schwartz et al. [11]. Universalism’s emphasis on understanding and tolerance of others and an emphasis on nature, is qualitatively different than the emphasis on caring and trust that underlies Benevolence, including the proposed new measure of general benevolence.

The differences between Benevolence and Universalism were highlighted by analyses of the 2016–17 European Social Survey reported in Nezlek [12]. In this study I found that Universalism was positively related to attitudes toward immigrants, refugees, and gays and lesbians, attitudes about the environment and climate change, social benefits, and income equality. In contrast, Benevolence was either unrelated or negatively related to these measures. The importance of this distinction was further confirmed by Nezlek [13]. In analyses of nine European Social Surveys (2002 through 2018) I found that endorsing Universalist values was negatively related to well-being, whereas endorsing Benevolent values was positively related to well-being.

In terms of adding items that measure benevolence toward “not close others,” the results of these two studies suggest that Universalism is not Benevolence directed toward a more general group. Although Universalism concerns the welfare of others, this concern is qualitatively different than the concern inherent in Benevolence. For example, Benevolence includes a desire for people to be seen as trustworthy by others. Such a desire is not part of Universalism. In both Nezlek [12] and Nezlek [13], I suggested that Universalism and Benevolence could be considered to be measures of Ideological and Interpersonal prosociality respectively, which are distinct, albeit related, constructs.

Consistent with Schwartz’s conceptualization of personal values, I assumed that the personal values that Schwartz described constitute the foundation for more domain-specific constructs such as SVO. Some indications about this correspondence can be found in the definitions presented in Table 1. For example, Benevolence, Universalism, and Conformity concern the feelings of others, whereas Hedonism, Achievement, and Power concern self-enhancement. The common focus of some of these basic values is indicated by the fact that Hedonism, Achievement, and Power are sometimes combined to create a higher-order factor named Self-enhancement, and Benevolence and Universalism are sometimes combined to create a higher-order factor named Self-transcendence.

Note: There is some inconsistency in how Hedonism has been treated by Schwarz and colleagues. For the PVQ-21, the measure used in the present study, Hedonism is located at the border of openness and self-enhancement but is scored as part of self-enhancement, e.g., Cieciuch et al. [14] A little differently, Schwartz and Cieciuch [10] in a study of the PVQ-RR (not the measure used in the present study), begin with the same assumption (Table 1, p. 1007), but Schwartz and Cieciuch then present some factor analyses suggesting that Hedonism should be modeled as part of openness to change. Regardless, whether Hedonism is part of Self-enhancement is not relevant to the present study because the present study did not examine higher-order factors.

### Previous research on relationships between SVO and personal values

Previous research has found that SVO and personal values are related, although the number of studies is limited. For example, Schwartz [15], a study of 90 university students who participated in a decomposed prisoner’s dilemma game, found that cooperation in the game was positively related to Universalism, Benevolence, and Conformity. In contrast, non-cooperation in the game was positively related to the importance of Power, Achievement, and Hedonism values.

Tao and Au [16] examined relationships between Schwartz’s Self-transcendence and Self-enhancement higher-order values and reward allocation in a dictator game among 257 university students. Similar to the results of Schwartz [15], they found that endorsing Benevolence and Universalism values were positively related to the awards allocated to others, whereas Achievement and Power values were negatively related to the awards allocated to others. Hedonism was not significantly related to reward allocation. Tao and Au also found that these relationships were stronger when either values or the self were primed.

A conceptually similar study to Tao and Au, Sagiv et al. [17], found similar, but not identical results to Schwartz [15] and Tao and Au [16]. The 46 students in Study 1 participated in a paired charity game. Contributions to charity (prosociality) were negatively related to Power, Achievement, and Hedonism, and were positively related to Universalism, Benevolence, and Tradition, but not to Conformity. In Study 2 (81 undergraduates who played a charity game), they found that Power was negatively related to cooperative behavior, whereas Benevolence was positively related. More important, in Study 2, Sagiv et al. manipulated the salience of values, and they concluded that “individuals’ values influenced their behavior in the Group Charity social-dilemma game” (p. 74), i.e., values were more of a cause of allocation of rewards than the reverse.”

Although informative, the existing research on relationships between SVO and personal values is limited in some ways. For example, the sample sizes have been small, which limits the power to detect relationships, particularly relationships involving different types of pro-selfs, who typically constitute a minority of participants. The sample in the present study was sufficiently large to provide adequate power to detect such differences.

### The present study

Participants in the present study completed the TDM and a measure of Schwartz’s personal values. Assuming that Benevolence and Universalism reflect prosocial values leads to the expectation that pro-socials will have higher scores than pro-selfs on these two measures. The same can be said for Conformity, which concerns taking other’s feelings into account when deciding how to behave. In contrast, for Hedonism, Achievement, and Power, which concern benefits for the self, pro-selfs should have higher scores than prosocials. These predictions are largely in line with the results of Schwartz [15], Sagiv et al. [17], and Tao and Au [16]. Guided by these expectations, the analyses examined differences among pro-socials, individualists, and competitors in the 10 basic values of Schwartz’s model of personal values plus the new measure of Benevolence created for the present study.

## Method

### Participants

The initial sample consisted of 1903 students (*M*_age_ = 18.9 years, *SD* = 1.02, range 18–28 years) who took an introductory psychology course at the College of William & Mary. They participated in partial fulfillment of a course requirement. Participation was voluntary; the course requirement could be fulfilled in ways that did not include participating in research. Data collection occurred over three semesters, the fall of 2002 (September through December of 2022), the spring of 2023 (January through April of 2023), and the fall of 2023 (September through December of 2023). Participants provided data using a secure website.

As explained below, the social value orientation of 226 of these 1903 participants could not be determined. Moreover, of the 1677 participants whose social value orientation could be determined, 22 did not answer any of the questions on the measure of personal values. Given this, the analyses included only those 1655 participants whose social value orientation could be determined and who answered some of the personal values questions. The number of participants included in each analysis is provided in the tables.

### Ethical statement

The study was conducted in accordance with the Declaration of Helsinki regarding the rights of research participants. Participants consented electronically by clicking on a link indicating their agreement to participate after being told that their names would not be associated with their answers and that they could terminate participation at any time without penalty. See supplemental materials for a copy of the consent procedure. Consistent with these instructions, responses were de-identified prior to analysis. The study was conducted over three semesters, and a separate ethics approval was obtained from the William & Mary Protection of Human Subjects Committee each semester. The protocol identifiers were: PHSC-2022-09-07-15836-ajbravo (approved 9/23/2022), PHSC-2023-02-01-16086-ajbravo (approved 2/13/2022), and PHSC-2023-09-13-16567-ajbravo (approved 9/18/2023).

### Measures

#### Social value orientation (SVO)

Social value orientation was measured using what is called the Triple Dominance Measure [TDM, 6]. Participants made nine decisions, in which they decided how many points to allocate to themselves and to a hypothetical other. Based on the choices they made, participants were classified as either prosocial, individualistic, or competitive. Consistent with past practice, participants were classified into a category if they made six choices in the same category. Participants who did not make six consistent choices were deleted from the analyses.

The instructions to participants were similar to those used in previous studies that have used the TDM. Participants were told:

“In this task we ask you to imagine that you have been randomly paired with another person, whom we will refer to simply as the "Other." This other person is someone you do not know and that you will not knowingly meet in the future. Both you and the "Other" person will be making choices by circling either the letter A, B, or C. Your own choices will produce points for both yourself and the "Other" person. Likewise, the other’s choice will produce points for him/her and for you. Every point has value: The more points you receive, the better for you, and the more points the "Other" receives, the better for him/her. Here’s an example of how this task works:

A You get 500 Other gets 100

B You get 500 Other gets 500

C You get 550 Other gets 300

In this example, if you chose A you would receive 500 points and the other would receive 100 points; if you chose B, you would receive 500 points and the other 500; and if you chose C, you would receive 550 points and the other 300. So, you see that your choice influences both the number of points you receive and the number of points the other receives. Before you begin making choices, please keep in mind that there are no right or wrong answers–choose the option that you, for whatever reason, prefer most. Also, remember that the points have value: The more of them you accumulate, the better for you. Likewise, from the "other’s" point of view, the more points s/he accumulates, the better for him/her. For each of the following nine choice situations, choose A, B, or C, depending on which column you prefer most. Provide your response by clicking on the button at the bottom of the column containing your answer.”

In this example, Choice A represents the competitive option, because it provides a larger difference between one’s own and the other’s outcomes (500–100 = 400) than either Choice B (500–500 = 0) or Choice C (550–300 = 250). Choice B represents the prosocial option because it provides a larger joint outcome (480 + 480 = 960) than provided by either of the other options, and Choice C represents the individualistic option because one’s own outcomes are larger than are those in Choice A or Choice B. A copy of the TDM is in the supplemental materials on the OSF site.

#### Personal values

Personal values were measured using the PVQ-21, a measure developed by Schwartz [18], with two additional items to measure Benevolence to people in general. These two items were: “It is important to him to be kind and caring to people, whether he knows them or not,” and “It is important to him that other people trust and can rely upon him. He wants people to know that he is trustworthy.” Compare these to the original two items, which focus on close others: “It is very important to him to help the people around him. He wants to care for their well-being,” and “It is important to him to be loyal to his friends. He wants to devote himself to people close to him.”

Prior to the study, the two new items measuring benevolence toward others were reviewed by a panel of three psychologists familiar with the measurement of individual differences including personal values. The two new items that were administered were judged to be measures of the intended construct of benevolence towards others, generally defined.

Note that the items measuring values are gender specific. This is done to enhance respondents’ involvement with the construct being measured. In the present study, participants indicated which pronouns they wanted to be used to display the items: male, female, or neutral (plural). A copy of the items used in the present study is available in the supplemental materials on the OSF site.

For each of the resulting 23 statements, participants indicated the extent to which a statement (e.g., to be rich, have money and expensive things; to be humble and modest, behave properly) described them using a 5-point scale with endpoints labelled 1 = *very much like me* and 5 = *not like me at all*. Before the analyses, responses to these items were reverse-scored so that higher numbers indicated stronger endorsement of a statement. Moreover, as recommended by Schwartz [18], responses were ipsatized, i.e., the mean response to the 23 items was subtracted from each response. This controlled for individual differences in the use of the response scale.

Responses to these 23 statements were combined to produce 11 scores representing the 10 basic values defined by Schwartz’s model: universalism, benevolence, conformity, tradition, self-direction, stimulation seeking, hedonism, achievement, power, security, and a score representing the new measure of benevolence to people in general. Researchers sometimes calculate what are referred to as higher-order factors which represent combinations of these basic values, e.g., Self-transcendence is a combination of Universalism and Benevolence. I chose not to do this because previous research has found that the basic values that comprise higher-order factors may be related to external criteria in different ways, e.g., Universalism and Benevolence are (respectively) negatively and positively related to well-being [13].

## Results

### Descriptive statistics and zero-order correlations

Based on their responses, 1683 of 1906 participants could be classified as either prosocial, individualistic, or competitive. The responses of 223 participants were not consistent enough to classify them into one of the three categories, and these participants were dropped from the analyses. Another 23 participants did not answer the values questions, and they were also dropped from the analyses. This left 1655 participants in the analyses: 1151 (69.5%) were classified as prosocial, 381 (23.0%) were classified as individualistic, and 123 (7.4%) were classified as competitive. For these participants, the mean number of prosocial choices for participants classified as prosocial was 8.49 (*SD* = .92), the mean number of individualist choices for participants classified as individualist was 8.28 (*SD* = 1.06), and the mean number of competitive choices for participants classified as competitors was 8.37 (SD = 1.02).

Correlations between personal value scores and descriptive statistics for these scores are presented in Table 2. Note that these are ipsatized scores, so the means are not the same as means based on the original 5-point scale. The correlation between the existing and new measures of benevolence was .526. Given that both of these measures concerned benevolence, a correlation of this size is not surprising. Nevertheless, it is important to keep in mind that such a correlation means that the two measures share only 28% of their variance, which leaves room for divergent validity.

**Table 2:** Descriptive statistics for, and correlations between, measures of personal values.

	*M*	*SD*	*n*	α	Bene	Bene-G	Conf	Trad	Sec	SelfD	Stim	Hed	Ach	Pow
Universalism	.469	.585	1653	.57	.272	.330	-.159	-.096	-.158	.048	-.102	-.206	-.276	-.432
Benevolence	.700	.585	1649	.68		.528	-.120	-.028	-.145	-.040	-.105	-.175	-.236	-.345
Benevolence-General	.805	.564	1647	.70			-.037	.030	-.085	-.074	-.155	-.231	-.181	-.368
Conformity	-.567	.903	1649	.61				.246	.191	-.349	-.406	-.318	-.138	-.053
Tradition	-.273	.814	1655	.30					-.021	-.182	-.171	-.290	-.323	-.172
Security	-.150	.781	1651	.49						-.251	-.409	-.114	-.005	-.038
Self-direction	-.012	.756	1653	.39							.146	-.058	-.074	-.142
Stimulation	-.218	.891	1652	.75								.187	-.106	-.075
Hedonism	-.203	.810	1652	.67									-.106	-.075
Achievement	.170	.764	1651	.68										.298
Power	.469	.585	1654	.41										

Note: For *n* = 1655: |*r*| > .049 significant at *p* < .05; |*r*| > .064 significant at *p* ≤ .01.

According to guidelines proposed by Shrout [19], six of these scales had moderate reliability (.61 to .80), three had fair reliability (.41 to .60), and two had slight reliability (.11 to .40). It must be kept in mind that with the exception of universalism, which consists of three items, all of these scales consist of two items, the minimum number of items to estimate reliability.

### Differences in values as a function of SVO

The primary analysis was a one-way ANOVA with SVO as the independent measure and the 11 basic value scores as the dependent measures. I also conducted two planned comparisons: one compared pro-socials and pro-selfs (pro-selfs consisted of individualists and competitors considered together) and a second compared individualists and competitors. According to G*Power [20], assuming α = .05, for a one-way ANOVA with three groups, a sample of 1655 provides power of .96 to detect a small effect (*f* = .10). For a t-test comparing pro-socials (*n* = 1151) with pro-selfs (*n* = 504), the power to detect a small effect (*d* = .2) was also .96. For a t-test comparing individualists (*n* = 381) with competitors (*n* = 123), the power to detect a medium effect (*d* = .5) was .99, the power to detect a small-medium effect (*d* = .35) was .92, and the power to detect a small effect (*d* = .2) was .49.

These analyses found a significant main effect for SVO in the analyses of universalism, both measures of benevolence, conformity, hedonism, achievement, and power. Moreover, for each of these measures, the planned comparison of pro-socials vs pro-selfs was significant. In contrast, the planned comparison of individualists vs. competitors found no significant differences in how strongly these two groups of participants endorsed any value, although the difference in the strength of endorsing benevolence values as defined by Schwartz originally approached the conventional level of .05 (*p* = .072).

The means for each group and the results of the main effects and the comparison of pro-socials and pro-selfs are presented in Table 3. This table also contains an estimate of the pooled *SD* based on the MS_*error*_ for each variable. Note that all of the significant main effects and significant differences between pro-socials and pro-selfs remained significant after controlling for false discovery rate [21].

**Table 3:** Results of analyses of variance and planned comparisons of basic values.

			Social value orientation			Main effect			Pro-social vs. pro-self		
	*n*	*SD*_*pool*_	Prosocial	Individual	Compete	*F*	*p*	*η* ^*2*^	*F*	*p*	*η* ^*2*^
Universalism	1653	.574	.543	.312	.262	31.71	.000	.037	55.76	.000	.033
Benevolence	1649	.581	.751	.558	.667	15.92	.000	.019	15.95	.000	.010
Bene-General	1647	.559	.854	.693	.692	14.62	.000	.017	23.48	.000	.014
Conformity	1649	.900	-.513	-.692	-.675	6.60	.001	.008	10.03	.002	.006
Tradition	1660	.814	-.260	-.314	-.264	< 1			< 1		
Security	1651	.780	-.176	-.080	-.120	2.27	.104	.003	2.67	.103	.002
Self-direction	1653	.756	-.011	-.018	.003	< 1			< 1		
Stimulation	1657	.891	-.243	-.171	-.134	1.54	.214		2.92	.088	.002
Hedonism	1652	.806	-.253	-.079	-.116	7.49	.001	.009	10.43	.001	.006
Achievement	1651	.760	.118	.316	.196	9.82	.000	.012	9.22	.002	.006
Power	1654	.806	-.633	-.287	-.243	34.43	.000	.040	58.40	.000	.034

Note: Bene-General refers to the new measure of benevolence. Column labeled *SD*_*pool*_ contains the square root of the mean square error.

Generally speaking, as can be seen from the means presented in Table 3, individualists and competitors (pro-selfs) tended to be more similar to each other than they were to pro-socials. Compared to pro-selfs, pro-socials endorsed Universalism and the two types of Benevolence values more strongly. Compared to pro-selfs, pro-socials endorsed Conformity, Hedonism, Achievement, and Power values less strongly.

Inspection of the means in Table 3 suggests that differences among the three TDM categories varied across the two measures of benevolence. Although a follow-up three (TDM category) by two (measure of Benevolence) ANOVA did not produce a significant interaction of category and measure, *F*(2,1643) = 2.36, *p* = .094, another analysis limited to individualists and competitors did find a near-significant interaction of category and measure, *F*(1,500) = 3.77, *p* = .053. This interaction was due to the fact that for the new measure, the difference between individualists and competitors was functionally 0 (.695 - .692 = .003), whereas for the existing measure, scores for individualists were lower than scores for competitors (.558 - .679 = -.121).

## Discussion

The present results suggest that social value orientation is related to more fundamental, basic personal values. Although the measure of SVO that was used, the TDM, produces three categories, prosocials, individualists, and competitors, the only distinction that mattered was between pro-socials and pro-selfs (individualists and competitors grouped together). This distinction has been found to be an important, perhaps the most important, distinction in previous research [22]. We discuss the casual precedence of personal values and social value orientation in a separate section below.

Consistent with the conceptualization of the pro-social orientation as reflecting a desire to equalize or maximize joint outcomes, pro-socials were higher than pro-selfs on three other-focused values, Universalism, which concerns treating people equally and fairly and includes concern for the environment. They were higher on the two measures of Benevolence, which concern more interpersonally focused values such as helping and supporting others and being trustworthy. Pro-socials were also higher than pro-selfs in terms of Conformity, another value focused on others. Pro-socials were lower than pro-selfs in terms of the self-focused values of Hedonism, Achievement, and Power.

### Levels of analysis of personal values and social value orientation

Schwartz’s various models all propose that personal values can be conceptualized at two levels of analysis, typically referred to as basic values and higher-order factors. For example, across these various models, Self-transcendence is defined as a higher-order factor consisting of the basic values of Universalism and Benevolence [e.g., 10]. Nevertheless, some research questions the validity of such higher-order factors because the basic values that comprise them are related to external criteria in different, sometimes opposing ways. For example, as discussed previously, Nezlek [13] found that Universalism and Benevolence were related to well-being in different ways, and Nezlek [12] found that Universalism and Benevolence were related to various social attitudes in different ways.

Regardless, the present results provide some support for the validity of these higher-order factors, at least in terms of the correspondence between basic values and social value orientation. Differences among the three categories in the strength of the endorsement of Universalism and of both the original and new measures of Benevolence were similar, suggesting that it was appropriate to think of these basic values as manifestations of the higher-order factor of Self-transcendence. Similarly, differences among the three categories in scores for Achievement and Power were similar, suggesting that it was appropriate to think of these basic values as manifestations of the higher-order factor of Self-enhancement. Pro-socials had lower scores on Hedonism than pro-selfs, which, as discussed in the introduction, is consistent with how Hedonism has been treated in some discussions of personal values, i.e., as a manifestation of Self-enhancement.

On the other hand, scores on Conformity, which is typically conceptualized as a manifestation of the higher-order factor of Conservation, varied across the three categories of SVO, whereas Tradition and Security, which are also typically conceptualized as manifestations of Conservation, did not. Moreover, although the main effect for SVO in the analysis of Security was marginally significant (*p* = .10), the mean differences were in the opposite direction to the mean differences in the analysis of Conformity. For security, pro-socials had lower (more negative) scores than pro-selfs, whereas for Conformity, pro-socials had higher (less negative) scores than pro-selfs.

### Expanding Benevolence to include people in general

Schwartz’s measure of Benevolence explicitly concerns benevolence toward close others, friends, family, and possibly acquaintance, but certainly not strangers. There is nothing inherently wrong in this. It was a choice, and the items reflect this choice. This focus of items measuring benevolence on close others is also readily apparent in other versions of Schwartz’s model such as the PVQ-RR, a 57 item scale [10].

The two items that were added to the PVQ-21 were similar to the original two items that measured benevolence in terms of the benevolent values they measured but differed in terms of the target of this benevolence. Although the results of the analyses of these two measures of benevolence were similar, follow-up analyses that treated the two measures of benevolence as a repeated measure found some support for the divergent validity of the new measure of benevolence. Consistent with sharing a pro-self orientation, individualists and competitors had near identical scores on the new measure, which focused on others in general. In contrast, individualists had lower scores than competitors (*p* = .07) on the original measure of Benevolence, which focused on close others.

At least in terms of the correspondence between personal values and social value orientation, the new measure appears to correspond more closely to SVO than the existing measure. This makes sense given the “generalized other” focus of the new measure. Nevertheless, this difference is not pronounced, and present results need to be considered as preliminary.

### Causal precedence

An important question in the study of relationships between personal values and social value orientation is the causal precedence of these constructs. Which is the cause, and which is the effect? Many discussions of SVO are moot on causal relationships between SVO and other dispositions. For example, the word “causality” does not appear in reviews by Thielmann et al. [2] and Murphy et al. [5]. Pletzer et al. [22] discuss causality, but their review is limited to relationships between SVO and cooperative behavior. Authors sometimes discuss how dispositions such as personality “predict” behavior in economic games [e.g., 23]. Although such language avoids direct claims of causality, typically, one thinks of predictors as causes and outcomes as effects.

Nevertheless, personal values as conceptualized by Schwartz and colleagues are meant to represent “broad motivational constructs that express what is important to people” [8], and the general logic of Schwartz’s model is that personal values serve as foundations for people’s behaviors, attitudes, and so forth. Such logic suggests that values are causes and the people’s choices in economic games are effects.

SVO as conceptualized in the present study (and many others) was inferred from the choices people made, i.e., it is a behavioral measure, and research has found that values lead to behaviors more regularly than behaviors lead to values. For example, in a series of six experiments, Verplanken and Holland [24] found support for a value-behavior causal relationship within the context of environmental values and environmentally-relevant behavior. Along the same lines, Sagiv and Roccas [25] reviewed the research on value-behavior relationships and proposed various mediators and moderators of such relationships. Nevertheless, the core of their analysis was that values lead to behaviors, not the opposite.

Although previous research suggests a causal relationship from values to social value orientation, the present study did not provide a basis to draw inferences about causality. The design of the present study was cross-sectional and static. Changes across time, which can be used to draw inferences about causality, were not examined.

It is possible that changes in SVO (a behavior) lead to changes in values, although I am unaware of any research demonstrating this. Although many researchers appear to assume a causal link from values to SVO rather than the reverse, often, the topic is not addressed explicitly. Clearly, resolving this issue requires collecting data that provides a basis for drawing inferences about causal relationships between personal values and social value orientation.

### The sample

The present study examined relationships between SVO and personal values among a sample of students, who compromise a sample of what is frequently described as “emerging adults.” The term was introduced by Arnett [26], and it refers to individuals who are between 18 years old and the mid-late twenties. Emerging adulthood is conceptualized as a transitionary stage between adolescence and adulthood. During emerging adulthood, individuals begin to formulate identities, which can include characteristics such as personal values and social value orientations.

Although emerging adulthood can be a time of exploration, it appears that personal values are relatively stable during emerging adulthood. For example, based on an eight-year study of the stability of values among a sample of 270 young adults, Vecchione et al. [27] concluded that: “The many transitions of young adulthood makes this a period with great potential for naturally occurring, long-term value change. However, our findings, which examine four different aspects of change, reveal the presence of substantial stability in basic personal values during this period” (p. 120). The stability of personal values during emerging adulthood may extend into adulthood per se, but this has not been established.

It should be noted that much of the research on SVO has examined student samples or young adults. For example, across the 770 studies Thielmann et al. [2] reviewed, the average age of 152,077 participants was 26.3 years, *Md* = 23.0, and most studies (72.8%) were conducted in the laboratory, suggesting the participants were university students. Although student samples may limit the generalizability of results in ways that samples of community members might not, in defense of this practice is the assumption that young adults do not differ from older adults in terms of what SVO represents. Nonetheless, this is an unexplored question that will require further research to answer.

### Strengths, limitations, and future directions

The present study had numerous strengths including the use of well-established measures of personal values and SVO. The results were clear and consistent with expectations and were consistent with how SVO and personal values have been conceptualized. The study also had a large sample, something that was particularly important in terms of comparing individualists and competitors. Competitors tend to be a relatively small minority of samples, typically 10–15% [e.g., 6] and sometimes lower, which means that without large total samples, comparisons of individualists and competitors can be underpowered. Competitors comprised 7.3% of the present sample, but there were 123 of them, which provided adequate power to detect differences between them and individualists (*n* = 385).

In terms of divergent validity, there were no differences as a function of SVO for basic values reflecting constructs that did not concern prosociality (or the lack thereof). These were: Tradition, Security, Self-direction, and Stimulation. Moreover, there was ample power to detect differences for these measures. The lack of differences for these basic values is important because like the other values, they are positively valent. The lack of differences in the analyses of these basic values suggests that SVO is not a manifestation of positive values in general; rather, it is a manifestation of values concerning the interpersonal domain in particular.

On the other hand, the study had shortcomings. For example, the study did not examine how relationships between values and SVO might vary as a function of situational affordances [2], nor did it examine how relationships might vary as a function of characteristics of the partners with whom participants are playing an economic game [22]. As mentioned previously, there is also the issue of the age of the sample. At this point, it would not be appropriate to speculate about how the present results might differ if affordances, relationships, or age of sample were considered. Examining such possibilities will require future research specifically designed to do so.

There is also the issue of “third variables,” variables that might explain, mediate, or confound the relationships found in the present study. There is little research or theory that suggests specific third variables, and the study was not designed to examine such possibilities, so it is difficult to speculate about this. Possible variables could include personality (e.g., agreeableness) and socio-political variables such as social dominance. Determining if such possibilities exist will require future research specifically designed to do so.

The main effects for SVO accounted for between 1% and 4% of the variance in measures of personal values. Such differences are fairly typical for research on SVO [2, 28]. According to guidelines discussed by Adams and Conway [29], an η^2^ of .01 represents a small effect, and an η^2^ of .06 represents a medium effect. Although such differences may not appear to be meaningful because they do not account for large amounts of variance, it is important to recognize that small effects can be important when they concern a large number of outcomes [30]. People probably make decisions about benefitting others vs benefitting themselves on an everyday basis, and the cumulative effect of values on these decisions (or vice versa) has the potential to be substantial.

Finally, there is the issue of the “disembodied” nature of the TDM. By design, the TDM asks respondents to imagine that they are making decisions in terms of someone they do not know and do not expect to meet. Moreover, in the TDM imaginary points are allocated. In contrast, research using other measures of SVO may involve pairing participants with another person (or persons) who is (are) identified and may involve the allocation of resources that have some value, e.g., tokens that can be redeemed for cash or other rewards. Research that has examined the correspondence of behavior in different economic games (including the TDM) suggests that different games provide similar descriptions of people [e.g., 31], but this is a relatively unexplored topic, and so it is not known if the use of the TDM limits the generalizability of the results of the preset study.

## Conclusions

The present results complement and extend existing research on relationships between personal values and social value orientation. Importantly, based on their content, values that were expected to vary as a function of SVO varied as expected, and values that were not expected to vary did not vary. Individuals with a pro-social orientation had higher scores than individuals with a pro-self orientation on Universalism, Benevolence, and Conformity values. In contrast, individuals with a pro-self orientation had higher scores than individuals with a pro-social orientation on Power, Achievement, and Hedonic values. Moreover, no differences were found between individualists and competitors for these values, and no differences of any kind were found for values that did not refer to the interpersonal domain, despite having adequate power to find such differences.

In terms of how personal values map onto social value orientation, the present results suggest that it may be more informative to conceptualize personal values in terms of basic values rather than higher-order factors. Although some results were consistent with conceptualizing values in terms of higher-order factors, there were enough results that did not, calling into question the utility of the higher-order factors for understanding SVO. Given this, in future research on relationships between personal values and SVO, it may be useful to conceptualize values in terms of basic values rather than higher-order factors, at least initially.
